# Distally based peroneal artery perforator-plus fasciocutaneous flap in the reconstruction of soft tissue defects over the distal forefoot: a retrospectively analyzed clinical trial

**DOI:** 10.1186/s13018-020-02019-4

**Published:** 2020-10-21

**Authors:** Ping Peng, Zhaobiao Luo, Guohua Lv, Jiangdong Ni, Jianwei Wei, Zhonggen Dong

**Affiliations:** 1 Department of Orthopaedics, The Second Xiangya Hospital, Central South University, No. 139 Renmin Road, Changsha, 410011 People’s Republic of China; 2 Department of Spine Surgery, The Second Xiangya Hospital, Central South University, Changsha, 410011 People’s Republic of China

**Keywords:** Ankle, Forefoot, Perforator flap, Peroneal artery, Surgical flaps

## Abstract

**Background:**

Distally based peroneal artery perforator-plus fasciocutaneous (DPAPF) flaps are widely used for reconstructing soft-tissue defects of the lower extremity. However, reports on the reconstruction of the defects over the distal forefoot using the DPAPF flaps are scarce. Herein, we describe our experience on the reconstruction of these defects using DPAPF flaps in a considerable sample size.

**Methods:**

Between February 2005 and August 2019, a total of 56 DPAPF flaps in 56 patients were used to reconstruct soft-tissue defects in the forefoot. In order to reduce the length of fascial pedicle and the total length of the DPAPF flaps, the ankles were fixed in dorsiflexion using a Kirschner wire before designing the flaps. The flaps were elevated by the anterograde–retrograde approach. Patient factors and flap factors were compared between the “survival” and “partial necrosis” groups.

**Results:**

Overall, 47 flaps had survived completely in one stage. Partial necrosis developed in nine flaps, with only one remnant defect covered using a local flap. By fixing the ankles in dorsiflexion, the length of the fascial pedicle was reduced approximately 2.35 ± 0.58 cm, the total length of the flap was simultaneously shortened by the same amount as the length of the fascial pedicle. The width of the fascia pedicle varied from 3.0 cm to 6.0 cm. The fascial pedicle width > 4 cm was found in 21 flaps. The partial necrosis rate of the DPAPF flaps with the top edge located in the 8th zone was significantly lower than that in the 9th zone (*p* < 0.05).

**Conclusions:**

The DPAPF flaps can be effectively used to reconstruct the defects over the distal forefoot because of convenient harvest and reliability. By fixing the ankle in dorsiflexion with Kirschner wire and widening the fascial pedicle appropriately, the top edge and LWR of the flaps will be decreased, and thus the procedures are helpful for the flaps survival.

## Introduction

Although modern medicine is evolving rapidly, covering soft-tissue defects in the lower extremities remains a clinical challenge. The reverse sural artery flap was first described by Masquelet et al. [1]. Free flap transfer and pedicle perforator flap transfer are currently widely performed. However, owing to the advantages such as reliability, simplicity, and no need for microvascular anastomosis, the distally based sural flap is an excellent option and widely utilized in reconstructive surgery for covering defects in the lower extremities [2–4].

Generally, the forefoot is defined as the area of the foot beyond the tarsometatarsal joints. The forefoot has the following unique peculiarities: the skin and soft tissues are thinner and non-elastic; the mobility is limited; it is important for ambulation; and it is difficult to perform vascularization in this area [5, 6]. In the present study, the distal forefoot was defined as the area beyond the midpoint of the metatarsal bones. After an open injury of the distal forefoot, the wound usually includes exposure of the tendons, joints, and bones. Clinical treatment is difficult through covering the defect in this area. The cross-leg flap [7, 8], other pedicled or local flap [5], and free flap [9] can be used to reconstruct the defect over the distal forefoot, but each flap has its respective indications and limitations. The cross-leg flap for that reconstruction of the defects in the region is a safe alternative; however, the patients who receive the cross-leg flap require additional nursing care and unavoidable reoperations, and face difficulties in daily activities. This will result in increased hospitalization costs, time to recovery, and psychological burden on the patients. Some pedicled or local flaps are appropriate to cover the small defects over the distal forefoot; but because of confined dimensions and the rotating arc, these flaps are difficult to satisfy the requirements of relatively larger regional repair. Free tissue transfer is an efficient way to reconstruct the defects over the distal forefoot, and it can be used to reconstruct larger defects as well; however, the procedure has some disadvantages, such as time limitation, increased technical complexity, and sacrifice of the main vessel. Amputation is sometimes needed in severe cases, which leads to great pain and shock to the patient’s mental and physical health. Some authors have reported successfully covering the defect in small patient populations over the distal forefoot using the distally pedicled sural fasciocutaneous flap [3, 6]. However, there are no reports on the reconstruction of these defects using the flap in a large sample size. Therefore, we describe our experience on the reconstruction of the defects over the distal forefoot using the distally based peroneal artery perforator-plus fasciocutaneous (DPAPF) flap in a considerable sample size.

## Methods

We retrospectively reviewed the cases of soft-tissue defect reconstruction over the distal forefoot using DPAPF flaps. Between February 2005 and August 2019, 56 patients underwent surgeries at our hospital. They provided written informed consent for publishing the treatment and follow-up data. Approval was obtained from the ethics committee of Central South University. The procedures used in this study adhere to the tenets of the Declaration of Helsinki.

Preoperative evaluation of the patients was performed, such as routine blood test, coagulation function test, electrocardiography, and chest plain radiography. Whether the patients had a serious cardiovascular disease, cerebrovascular disease, diabetes mellitus (DM), or peripheral vascular disease (PVD) was also evaluated. All the patients were able to tolerate the operation. Color Doppler ultrasonography was performed to assess the quality of the vessel in the lower extremities and mark the location of the peroneal artery perforator. We excluded patients in whom the defects were due to DM or PVD. We also excluded those in whom the defects were repaired using a perforator pedicle-based sural flap.

The possible risk factors [10]—including patient factors (age, sex, etiology of the defect) and flap factors (pivot point site, length and width of the fascial pedicle, length and width of the skin island, total length of the flap, length-width ratio [LWR], and top-edge location of the flap)—for partial necrosis of the flaps were analyzed. The posterior aspect of the calf was divided into nine zones to locate the top edge of the flap [10]. In patients with more than 6 months of follow-up postoperatively, the reconstruction outcomes of the flaps were assessed in terms of pain, appearance, footwear restrictions, functional restrictions, and patient satisfaction, which were described by Boyden et al [11].

### Operative technique

**Fig. 1:**
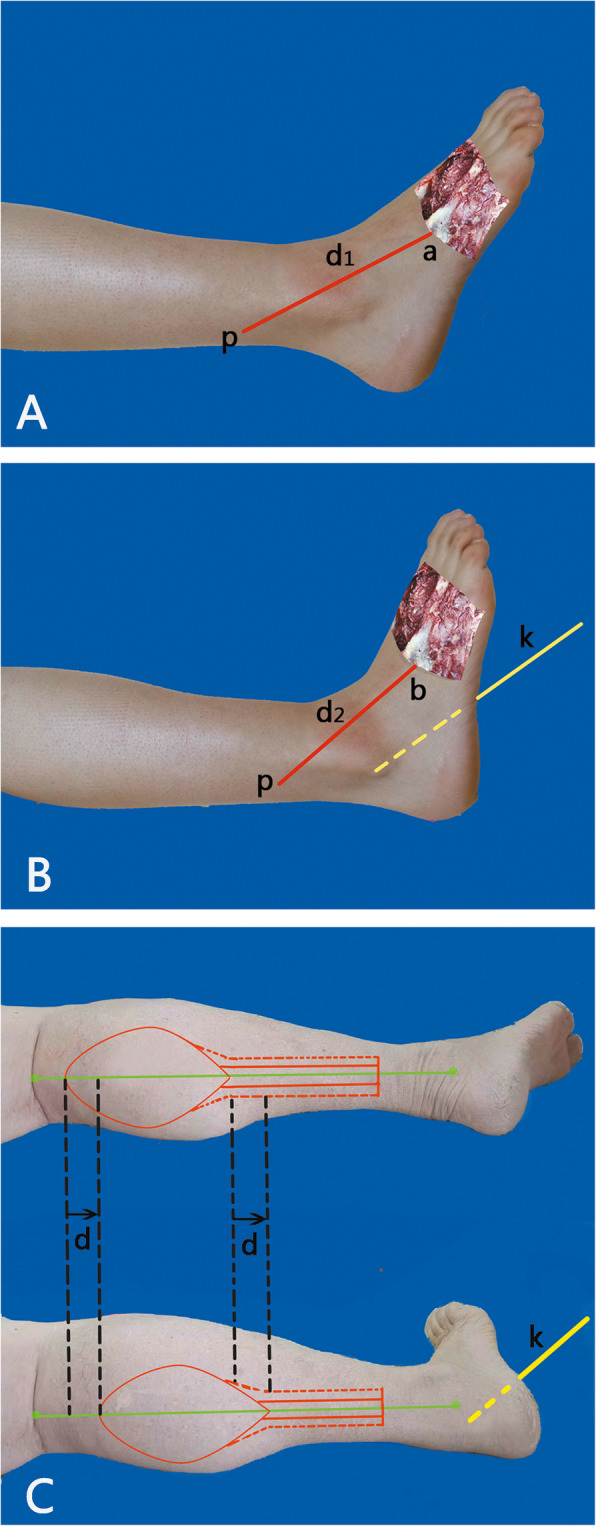
Schematic diagram of the DPAPF flap design in the two conditions. **a** The ankle is in a relaxed state. **b** The ankle is fixed in dorsiflexion using a Kirschner wire. The distance between the pivot point and the recipient area was shortened. **c** When the ankle is fixed in dorsiflexion using a Kirschner wire, the length of the fascial pedicle was reduced by “*d*” cm, and the total length of the flap also reduces by “*d*” cm. p, pivot point; point a or point b is where the defect is closest to the pivot point; d_1_ and d_2_ represent the shortest distances from the pivot point to the defect. *d* = *d*_1_−*d*_2_. K, Kirschner wire

Thorough debridement is the most important step in the management of such defects. The patients were advised smoking cessation since admission to the hospital. Before designing the flap, the ankle was fixed in dorsiflexion using a Φ2.0 or Φ3.0 Kirschner wire. The procedure was helpful in shortening the distance from the pivot point to the recipient area, which can reduce the length of the fascial pedicle and the total length of the DPAPF flap (Fig. 1). In order to avoid affecting the function of the ankle, the Kirschner wire would be removed at 3 weeks after the operation.

The flaps were harvest by the “anterograde–retrograde” approach, which was described detailly in our previous paper [12]. The patient was placed in the lateral position. The axial line of the flap was drawn from the midpoint between the posterior border of the fibula and the lateral border of the Achilles tendon to the midpoint of the popliteal fossa. The pivot point was designed to be at the level of the perforator which was marked by color Doppler ultrasonography pre-operation. The length of the fascial pedicle was approximately 2 cm longer than the nearest distance between the pivot point and the defect, and was marked at the axial line. The width of the fascia pedicle varied from 3.0 to 6.0 cm. In order to decrease the compression of the subcutaneous tunnel and the pedicle, a skin strip was designed overlying the fascial pedicle, and the width was about 2.0 cm. Skin island of the flap was designed along the axial line, approximately 1.0 cm larger in periphery than the form of the defect. After designing the DPAPF flap, the perforator located at the pivot point was explored firstly. Once the perforator located at the pivot point was ensured, the flap was raised subfascially from top to bottom. If the identified perforator was not located nearby the planned pivot point, the flap should be redesigned. The passage from the pivot point to the defect was incised through the lateral approach. All the donor sites of the DPAPF flaps were resurfaced with skin grafts. Postoperatively, in order to avoid compression and promote the venous drainage of the flaps, elevation of the extremity was performed for 1 week.

### Statistical analysis

All the data were analyzed by 2 authors (PP and ZBL) using the statistical software package SPSS (Version 17.0; SPSS Inc., Chicago, IL). Data of the continuous variables were analyzed using Student’s *t* test and expressed as mean ± standard deviation. The categorical data were analyzed using Fisher’s exact test and expressed as a constitute ratio. *P* < 0.05 was considered to indicate statistical significance.

## Results

Of the 56 flaps, 47 flaps survived uneventfully, partial necrosis was observed in nine (16.1%) cases. There was no case of complete necrosis. Six remnant defects were successfully repaired using skin grafting, and in two other cases, the remnant defects were repaired with secondary suturing. The only remnant defect (1.8%) was repaired using a local flap.

The average age of the patients was 37.4 years (range, 2-81). The defects were mainly caused by trauma (87.5%, 49/56), while the non-traumatic causes included chronic osteomyelitis (*n* = 6) and soft-tissue tumor (*n* = 1, well-differentiated squamous cell carcinoma, T1N0M0). The average elevation time for the DPAPF flaps was approximately 33 min. The mean length and width of the fascial pedicle were 10.26 ± 3.98 cm (range, 2.5-18) and 4.28 ± 0.54 cm (range, 3-6), respectively. The fascial pedicle width > 4 cm was found in 21 flaps. The mean length and width of the skin island were 13.13 ± 3.51 cm (range, 7.5-22) and 8.94 ± 1.73 cm (range, 5.5-13), respectively. The mean total length of the flaps was 23.38 ± 4.22 cm (range, 12.5-28), and the mean LWR of the flaps was (5.49 ± 0.93):1 (range, [3.13-6.88]:1). The dimension of the skin island ranged from 8 cm × 5.5 cm to 22 cm × 13 cm. The constitute ratios of patient factors (Table 1) and continuous variables of the DPAPF flaps (Table 2) displayed no significant differences between the survival and partial necrosis flap groups (*p* > 0.05).

**Table 1:** Demographic and clinical characteristics of the patients in both groups

Variable	Survival flaps (*n* = 47), *n* (%)	Partial necrosis flaps (*n* = 9), *n* (%)	p*
Age (years)			0.725
≤ 40	25 (86.2)	4 (13.8)	
> 40	22 (81.5)	5 (18.5)	
Sex			0.385
Male	38 (86.3)	6 (13.6)	
Female	9 (75.0)	3 (25.0)	
Etiology of the defect			0.583
Trauma	40 (81.6)	9 (18.4)	
Non-traumatic	7 (100.0)	0 (0.0)	

^*^Fisher’s exact test

**Table 2:** Comparisons of continuous variables in both groups

Parameters^a^	Survival flaps (*n* = 47)	Partial necrosis flaps (*n* = 9)	*t*	*p*
Fascial pedicle (cm)				
Length	10.52 ± 3.86	8.89 ± 4.57	1.130	0.264
Width	4.30 ± 0.57	4.17 ± 0.35	0.666	0.508
Skin island (cm)				
Length	13.06 ± 3.61	13.44 ± 3.14	−0.295	0.769
Width	8.87 ± 1.67	9.28 ± 2.06	−0.639	0.526
Total length (cm)	23.59 ± 4.18	22.33 ± 4.52	0.813	0.420
Length-to-width ratio	5.52 ± 0.92	5.36 ± 1.02	0.464	0.645

^a^The values are expressed as mean ± standard deviation

The pivot points of the DPAPF flaps were located at 3.5-9.0 cm above the tip of the lateral malleolus. LWR ≥ 5:1 and skin island width ≥ 8 cm were found in 82.1% (46/56) and 76.8% (43/56) of the overall DPAPF flaps, respectively. The constitute ratios of these indicators did not show any significant difference (*p* > 0.05) between the partial necrosis and survival flap groups (Table 3).

**Table 3:** Comparisons of the constituent ratios of pivot point, length-width ratio (LWR), width of the skin island, and top-edge location

	Survival flaps (*n* = 47), *n* (%)	Partial necrosis flaps (*n* = 9), *n* (%)	p*
Pivot point (above the tip of the lateral malleolus)			1.000
≤ 7 cm	41 (83.7)	8 (16.3)	
> 7 cm	6 (85.7)	1 (14.3)	
Length-to-width ratio			0.656
< 5:1	8 (80.0)	2 (20.0)	
≥ 5:1	39 (84.8)	7 (15.2)	
Width of the skin island			1.000
< 8 cm	11 (81.8)	2 (18.2)	
≥ 8 cm	36 (83.7)	7 (16.3)	
Top-edge location			0.005
8th zone	35 (94.6)	2 (5.4)	
9th zone	12 (63.2)	7 (36.8)	

^*^Fisher’s exact test

Without fixation of the ankle in dorsiflexion, there would be 10 DPAPF flaps with the top edge lying above the popliteal fossa crease, and the top edge of the remaining most flaps would locate in the 9th zone. However, by fixing the ankles in dorsiflexion, the length of the fascial pedicle was reduced approximately 2.35 ± 0.58 cm (range, 1.5-3.5), the total length of the flap was simultaneously shortened by the same amount as the length of the fascial pedicle. In the present study, the top edge of all the flaps was located in the 8th zone (66.1%, 37/56) or the 9th zone (33.9%, 19/56) of the calf. Partial necrosis rate of the DPAPF flaps in the 8th zone was significantly lower than that in the 9th zone (*p* = 0.005) (Table 3).

**Fig. 2:**
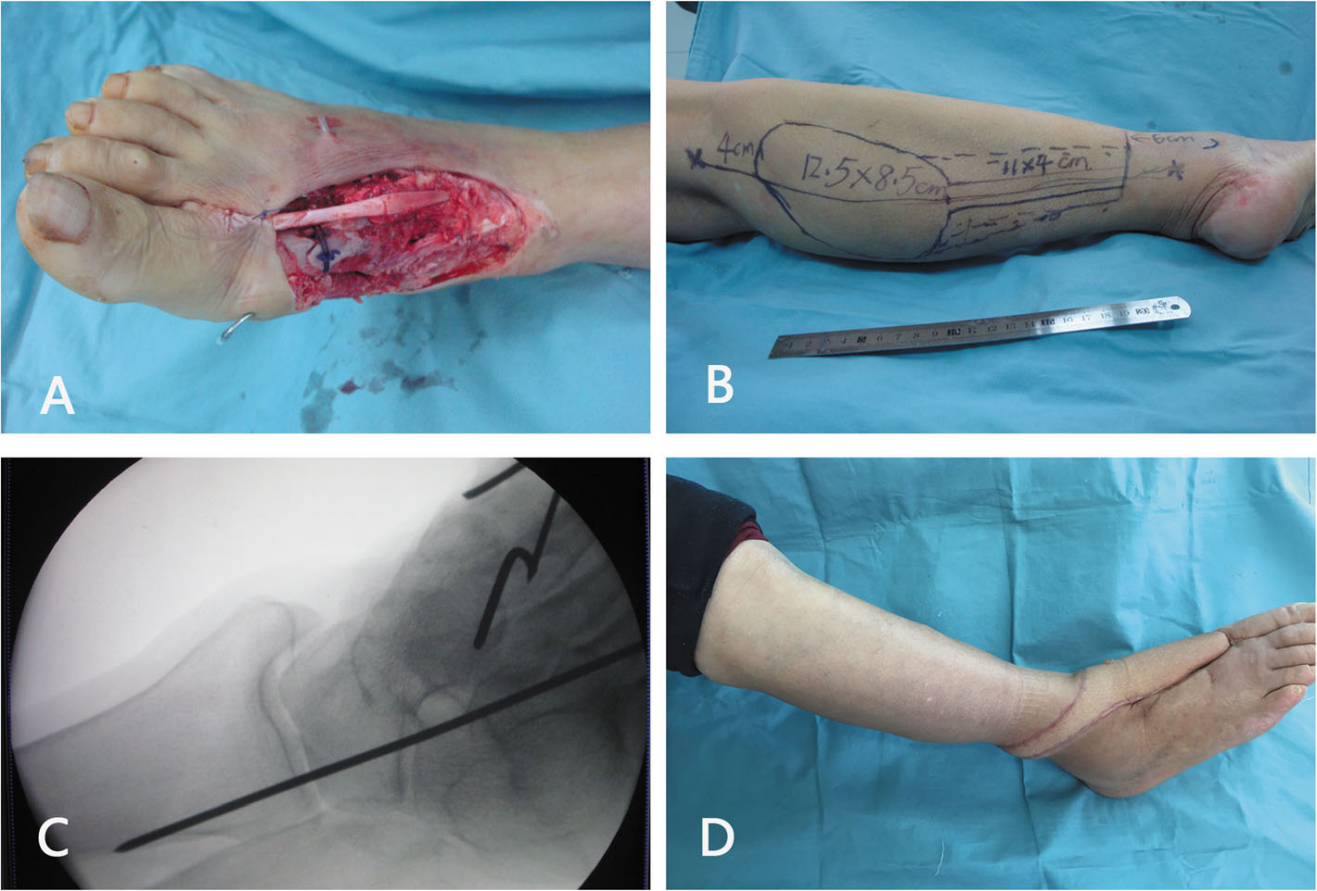
A 63-year-old woman had a traumatic soft-tissue defect over the distal forefoot. **a** Image of the defect after thorough debridement. The most distal part of the defect was beyond the midpoint of the metatarsal bone, which required reconstruction. **b** Design of a DPAPF flap. **c** Intraoperative fluoroscopy confirmed that the ankle was fixed in dorsiflexion with a Kirschner wire. **d** Outcomes of the flap at 17 months postoperatively

**Fig. 3:**
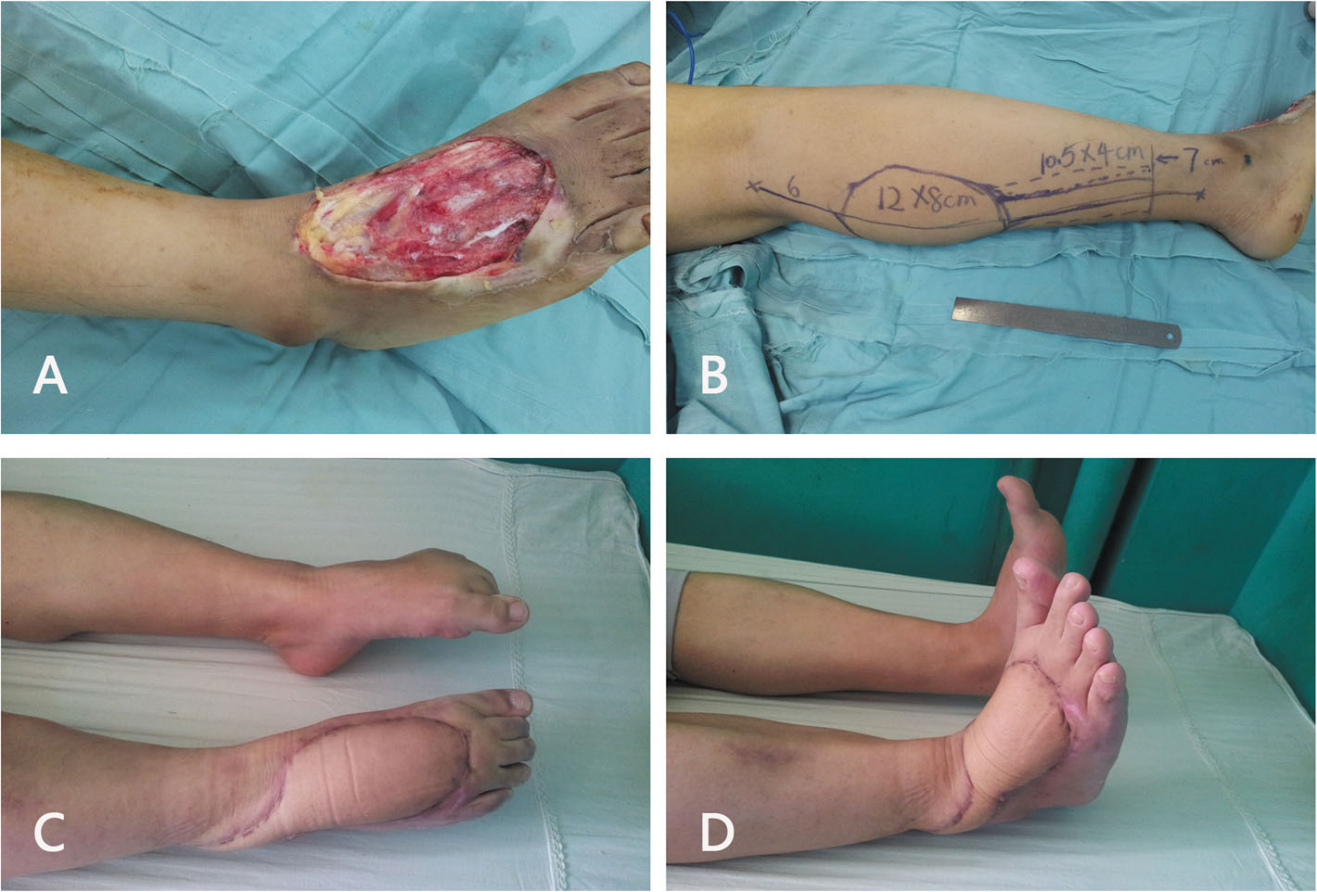
A 32-year-old man had a traumatic soft-tissue defect over the distal forefoot. **a** Image of the defect after thorough debridement. The most distal part of the defect was beyond the midpoint of the metatarsal bone, which required reconstruction. **b** Design of a DPAPF flap. **c** and **d** Appearance and reconstruction outcomes at 13 months postoperatively

During a mean postoperative follow-up of 17.2 months (range, 2 weeks-119 months), no infection or skin necrosis was observed at the donor or recipient sites. None of the patients complained of the loss of sensation on the lateral aspect. There were no neurotrophic ulcers. There were no cases of unacceptable pain in the ankle, and the function of the ankle was not affected. There were 48 (85.7%) flaps with more than 6 months of follow-up postoperatively. The reconstruction outcomes of the 48 flaps were evaluated, which were excellent in 38 cases, good in eight cases, and fair in two cases (Figs. 2 and 3).

## Discussion

Distally based peroneal artery perforator-plus fasciocutaneous flap does not only have the characteristics of the distally pedicled sural fasciocutaneous flap but also has the unique characteristics of dual blood supply and venous outflow from the perforator and fascial pedicle [13, 14]. However, the reliability of these flaps remains the main concern. The highest necrosis (including complete and partial) rate of the distally based sural flap was 35.7% (25/70) [15]. In the last decade, various necrosis rates of the distally based sural flap in studies with relative large sample sizes (*n* ≥ 40) were reported to be 3.9% (2/51) by Asʼadi et al. [16], 8.3% (13/156) by Gill et al. [4], 8.8% (9/102) by Dhamangaonkar et al. [17], 11.8% (9/76) by Herlin et al. [18], 17.2% (15/87) by Raza et al. [19], 20.5% (9/44) by Dai et al. [20], 22.3% (33/148) by Schmidt et al. [21], 30.6% (36/85) by Perumal et al. [22]. In the present study, the sample size was relatively large and there were no cases of complete necrosis of the flap. Forty-seven (83.9%) flaps completely survived within one stage, while the other eight (14.3%) defects were reconstructed using the DPAPF flaps with simple skin grafting or suturing in the second stage, only one (1.7%) remnant defect was covered with a local flap. The results suggest that the DPAPF flaps are relatively reliable for reconstructing the defects in the distal forefoot. Once partial necrosis of the flap occurred, the remnant defects in most cases were covered successfully with a simple procedure. The DPAPF flap is particularly suited for the reconstruction of the defects over the distal forefoot for physicians who are not experienced with using free flaps or patients who are not eligible for undergoing reconstruction with a free flap [23, 24].

The defects over the distal forefoot are more distal than those over other parts of the foot. Covering the soft tissue defects in this region is difficult. Zgonis et al. and Gözü et al. utilized the cross-leg reverse sural artery flap to reconstruct the defects over the distal forefoot in the small sample size [7, 8]. However, it is not an optimal choice because the prolonged postoperative immobilization of both lower limbs and the unavoidable reoperations is a great inconvenience for patients. Zhu et al. reconstructed the defect over the forefoot using 5 types of free flaps for 41 patients, and two flaps were lost by repeat exploration [9]. Free flap can be used to reconstruct larger defects; however, the procedure has some disadvantages, such as time limitation, requirement of additional equipment, increased technical complexity, sacrificing of the main vessel, trained microsurgeons and teams, postoperative monitoring requirements, and the need for a relatively longer learning curve, and there is always a risk of reexploration.

When the DPAPF flap is designed to reconstruct the defect over the distal forefoot, the contour and location of the defect in every case are invariable. Thus, the length of the fascial pedicle and total length of the flap are subject to the height of the pivot point. The higher the pivot point is, the longer is the fascial pedicle and the flap needed. Li et al. considered that reverse sural neurofasciocutaneous flaps with the pivot point of 5–7 cm above the lateral malleolus were not able to repair middle and distal foot injuries, and they advised that the defects in the region should be covered with the flaps with lower pivot points [24]. By the anatomical study, Zhang et al. found the length and the outer diameter of the perforator located at the lower pivot point were shorter and smaller, and the perforator can still nourish a sizeable distally based flap for foot and ankle coverage [25]. However, the length and diameter of the perforator located at 5-7 cm above the tip of lateral malleolus are larger, which is helpful for avoiding the excessive torsion of the perforator and the flap blood supply.

Based on our previous study, partial necrosis rates of reverse sural artery flaps increased significantly when the LWR was 5:1 or more, skin island width was 8 cm or more, or the top edge of the flap was located in the 9th zone [10]. In the current study, the proportions of the aforementioned first two unfavorable conditions were 82.1% and 76.8%, respectively; the top edge of the flaps was located in the 8th zone (66.1%) or the 9th zone (33.9%); the mean of length of fascial pedicle and skin island, total length, and the LWR were more than those parameters which reported by Wei et al. [10]. Despite so many unfavorable conditions, the survival rate of the DPAPF flaps was relatively higher, and the only remnant defect (1.8%) was repaired using a local flap. The outcome of the DPAPF flaps for repairing the defects over the distal forefoot was acceptable. The reason why the DPAPF flaps can survive longer and more reliably is that an average of 3.2 true anastomoses connects the perforators without change in the caliber on the posterior calf, which are parallel to the sural nerve [26]. Compared with the survival flaps, the constitute ratios of the two indicators (LWR of 5:1 or greater and skin island width ≥ 8 cm) were not significantly different in the partial necrosis flaps (*p* > 0.05). The partial necrosis rate of the DPAPF flaps located in the 8th zone (5.4%) was significantly lower than that of the flaps located in the 9th zone (36.8%) (*p* < 0.05). The finding suggests that the DPAPF flaps with the top edge in the 8th zone were safer and more reliable in repairing the defects of the distal forefoot. It is consistent with both our previous research [10] and the “Surgical Unsafe Zone” reported by Mojallal et al. [27].

As mentioned above, the partial necrosis rate of the DPAPF flaps was most associated with the top edge of the flap. By fixing the ankles in dorsiflexion, in the setting of invariable the skin island and the pivot point, the length of the fascial pedicle was reduced about 2.35 cm on average, and the total length of the flap was simultaneously shortened by the same amount. Meanwhile, the top edge of the flaps was descended by the procedure; otherwise, the proportion of flaps with the top edge in the 9th zone would be higher, even the proximal border of some flaps would beyond the popliteal crease. The external fixation device is a device that prevents complications of distally based sural flaps and facilitates postoperative care [15, 28]. However, it requires an additional nail for external fixation nursing care during the postoperative fixation period, and it increases the financial burden on the patients. Pallua et al. introduced the concept of reconstructing the defects in the distal forefoot using pedicle flaps and fixing the feet using a cast [5]. We did not use this approach because of difficulties in changing the dressings and the high risk of pressure sores of the heel. In the present study, the ankle was fixed in dorsiflexion using a Kirschner wire, which is cheaper and requires a more convenient dressing and nursing care. There was no case of infection of the ankle or nail. After 3 weeks of the operation, the Kirschner wire was pulled out after confirming the stability of the flaps. The period of ankle fixation was short; therefore, the function of the ankle was not affected.

Width of fascial pedicle is associated with arterial supply [1] and venous drainage [29]. When the DPAPF flap was utilized to repair the defect over the distal forefoot, if the dimension of the skin island was larger, the fascial pedicle should be widened appropriately. This tip was also helpful for reducing the LWR to prevent complications of the flaps to some extent.

The mean duration of DPAPF flap elevation was approximately 30 min, which highlights that the flap can be elevated easily and quickly, and the defects over the distal forefoot can be covered without microsurgical techniques and sacrificing major arteries of the lower extremities. The disadvantage was that the donor sites were closed by resurfacing with a skin graft, which may be related to the relatively larger dimensions of the defects. In the present study, although a follow-up of 8 weeks or 3 months was enough [11, 20], we evaluated the reconstruction outcomes of the DPAPF flaps over more than 6 months of follow-up. The reconstruction outcomes were very satisfactory with only 2/48 cases having evaluated fair outcomes.

To the best of our knowledge, this is the first study to introduce the reconstruction of the defects over the distal forefoot with DPAPF flaps with the largest number of patients. A randomized controlled trial was not possible because this study was a retrospective review, which is a major limitation of the study. Further studies with larger sample sizes are required to improve the success rate of DPAPF flaps in the reconstruction of defects over the distal forefoot.

## Conclusions

Distally based peroneal artery perforator-plus fasciocutaneous flaps can be effectively used to reconstruct the defects over the distal forefoot because of convenient harvest and reliability. By fixing the ankle in dorsiflexion with Kirschner wire and widening the fascial pedicle appropriately, the top edge and LWR of the flaps will be decreased, and thus the procedures are helpful for the flaps survival.

## Data Availability

Not applicable

## Abbreviations

DPAPF: Distally based peroneal artery perforator-plus fasciocutaneous
DM: Diabetes mellitus
PVD: Peripheral vascular disease
LWR: Length-to-width ratio
